# Mpox: an old disease poses a new threat

**DOI:** 10.2471/BLT.24.292707

**Published:** 2024-12-01

**Authors:** Nicaise Ndembi, Moréniké Oluwátóyìn Foláyan, Salim S Abdool Karim

**Affiliations:** a Africa Centres for Disease Control and Prevention, Haile Garment Square, PO Box 3243 Addis Ababa, Ethiopia.; b Department of Child Dental Health, Obafemi Awolowo University, Ile-Ife, Nigeria.; c Centre for the AIDS Programme of Research in South Africa, Nelson R Mandela Medical School, University of KwaZulu-Natal, Durban, South Africa.

Since mpox was first described in humans in 1970 in the Democratic Republic of the Congo, occasional cases or small outbreaks in African countries have occurred, usually resulting from animal-to-human transmission.^1^ The virus primarily spreads through direct contact with infected bodily fluids, lesions or respiratory droplets, making person-to-person transmission common.

In 2022, an outbreak of clade IIb variant of mpox, which began in Nigeria, spread in 110 countries and territories globally. Most cases were reported in Europe and the Americas, mostly in men who have sex with men.^1^ The spread led the World Health Organization (WHO) to declare mpox a Public Health Emergency of International Concern, later lifted in May 2023.^1^^,^^2^

A new mpox variant labelled clade Ib is now spreading rapidly in the Democratic Republic of the Congo and three neighbouring countries (Burundi, Central African Republic and Rwanda), and in August 2024 the Africa Centres for Disease Control and Prevention and WHO again declared mpox a Public Health Emergency of Continental Security and International Concern, respectively.^3^ Phylogenetic studies conducted in September 2023 indicated that a new variant was spreading in the mining region of eastern Democratic Republic of the Congo.^4^ Sex workers constituted about one third of the people diagnosed with infection, suggesting that the new variant may be also spreading through sexual contact.^5^

Sexual transmission presents challenges for containment efforts because related stigma and fear of discrimination can discourage timely care and disclosure, as seen in early outbreaks of human immunodeficiency virus (HIV). Delay in accessing health services prolongs transmission within communities. Close skin-to-skin contact during sexual activity facilitates viral spread, especially when lesions are in hard-to-see areas. Mild or atypical symptoms may be mistaken for other sexually transmitted infections, complicating early diagnosis and allowing continued transmission. Mpox also spreads rapidly within tight-knit sexual networks, where multiple partners create a high-risk environment for ongoing infection cycles, similar to HIV.^6^

Containing an mpox outbreak in Africa requires coordinated action from public health institutions. WHO plays a key role in global coordination, while the Africa Centres for Disease Control and Prevention mobilizes resources and coordinates the response for the continent.^7^ In countries, health ministries and health workers are critical for curbing the spread of the virus. All actors are working under a unified strategy. The One Team, One Plan, One Budget and One Monitoring and Evaluation Framework is a promising start.^7^

Clear, stigma-free public health messaging targeting high-risk populations is essential. Lessons from HIV responses emphasize the importance of culturally sensitive education that promotes early detection, safer sexual practices and reducing the number of partners.^8^ Expanding access to rapid testing and vaccination for at-risk groups is crucial, with vaccines made available without stigmatizing specific populations.

While more research is required to understand the new variant and develop appropriate diagnostics, vaccines and therapeutics, scaling up testing for surveillance and case detection is also essential. Plans are underway to deploy over 10 000 community health workers in the Democratic Republic of the Congo to engage with communities to enhance referral to health-care services and detection of cases.^9^ Anonymous partner notification for cases transmitted through sexual contact, can help identify, test and trace at-risk individuals. Integrating mpox prevention into existing sexual health services could further reduce barriers to care while offering comprehensive treatment.

Other lessons from the global HIV response can inform efforts to curb sexual transmission of mpox.^10^ The detrimental impact of stigma, the need to involve affected communities and engage community leaders, the need for inclusive public health messaging and sustained investment in public health infrastructure, research and prevention strategies are relevant considerations for current and future epidemics.

The most important lesson from the HIV response, however, is that global solidarity is essential. Collaboration between countries, institutions and communities facilitates knowledge and data sharing, and enhances coordination. Building new partnerships can support greater resource mobilization. As with HIV, a united effort that prioritizes equity, inclusivity and sustained support will be key to overcoming stigma, improving access to care and achieving long-term control of a disease that is partly driven by sexual transmission.
